# Effects of *Bacillus subtilis* ZJ-2019-1 on Zearalenone Toxicosis in Female Gilts

**DOI:** 10.3390/toxins13110788

**Published:** 2021-11-08

**Authors:** Junnan Zhang, Yunduo Zheng, Hui Tao, Jie Liu, Peng Zhao, Fan Yang, Zonghao Lv, Jinquan Wang

**Affiliations:** 1 Key Laboratory of Feed Biotechnology, Ministry of Agriculture and Rural Affairs, Feed Research Institute, Chinese Academy of Agricultural Sciences, No. 12 Zhongguancun South Street, Beijing 100081, China; zhangjunnan@caas.cn (J.Z.); zhengyunduo@caas.cn (Y.Z.); taohui@caas.cn (H.T.); liujie05@caas.cn (J.L.); zhaopeng02@caas.cn (P.Z.); yangfan03@caas.cn (F.Y.); 2 Animal Husbandry Department, Agricultural Science Research Institute, Huaihua 418000, China; lvzonghao@caas.cn

**Keywords:** zearalenone, *Bacillus subtilis* ZJ-2019-1, reproduction, gilts

## Abstract

The purpose of this research was to investigate the toxicity of zearalenone (ZEN) on the growth performance, genital organs, serum hormones, biomarkers, and histopathological changes of female gilts and to evaluate the efficacy of *Bacillus subtilis* ZJ-2019-1 in alleviating ZEN toxicosis in gilts. Twenty-four female gilts were randomly allocated to four groups with six replicates per group and one gilt per replicate, fed on four feeds prepared previously, which were basic diet (control group, C group), ZEN diet (Z group), Zlb diet (Zlb group) containing B. subtilis ZJ-2019-1 in liquid form, and Zdb diet (Zdb group) containing *B. subtilis* ZJ-2019-1 in dehydrated form. The results showed that the vulva size and relative weight of reproductive organs had no significant difference in the control group, Zlb group, and Zdb group, but were significantly lower than in the Z group (*p* < 0.05); the relative weight of the liver was lower in the C group, Zlb group, and Zbd group than in the Z group (0.05 < *p* < 0.1). The concentration of serum glutamate dehydrogenase (GLDH) was lower, but follicle-stimulating hormone (FSH) was higher in the Z group, Zlb group, and Zdb group than in the Z group (0.05 < *p* < 0.1). Additionally, serum luteinizing hormone (LH) concentration had no significant difference in the C group, Zlb group, and Zdb group but was significantly lower than in the Z group (*p* < 0.05); estradiol (E2) was significantly lower in the Zlb group and Zdb group than that in C group, but significantly higher than that in Z group (*p* < 0.05); PRL was significantly higher in the Zlb group and Zdb group than in the C group, but was significantly lower than in Z group (*p* < 0.05). ZEN and its reduced metabolites were measured in biological samples after enzymatic hydrolysis of the conjugated forms. The concentration of serum ZEN and its metabolite, α-zeralenol (α-ZOL), had no significant difference in Zlb, Zdb, and control groups but was significantly lower than in the Z group (*p* < 0.05); urine ZEN and its metabolites, α-ZOL and β-zeralenol (β-ZOL), had no significant difference in Zlb, Zdb, and control groups but was significantly lower than in the Z group (*p* < 0.05). Cell damages were observed in the liver, uterus, and ovary of gilts in the Z group and alleviated in Zlb and Zdb groups, but the loss of oocytes was irreversible in the ovary. The ZEN-contaminated diet caused serious changes in female hormones and brought harm to the livers and reproductive organs, but *B. subtilis* ZJ-2019-1 could naturally remove the ZEN significantly, which ameliorated the reproductive impairment in gilts caused by ZEN. The addition of *B. subtilis* ZJ-2019-1 to ZEN-contaminated feeds could ameliorate the toxic effects effectively, regardless of liquid or dry culture. Therefore, the *B. subtilis* ZJ-2019-1 strain has great potential industrial applications.

## 1. Introduction

Mycotoxin-contaminated food/feed is a health risk worldwide. Aflatoxin B_1_ (AFB_1_) is the most potent carcinogenic mycotoxin in humans, and ochratoxins, fumonisins, trichothecenes, and zearalenone also have potential health risks [1,2]. As one of the most prevalent non-steroidal estrogenic mycotoxins consisting of 6-(10-hydroxy-6-oxo-trans-1-undecenyl)-β-isophthalic lactone, zearalenone (ZEN) is mainly produced by various strains of the genus *Fusarium*, primarily *Fusarium graminearum*, *Fusarium culmorum*, and *Fusarium cerealis* [3,4]. Moreover, as one of the most frequently detected mycotoxins in animal feed, ZEN can be metabolized and deposited in meat, eggs, milk, and other animal products, which seriously endangers the health of livestock and poultry when they are exposed to it, in most cases through their feed, and even threatens human food safety and health when humans consume products from these animals [5]. ZEN can accumulate in moldy crops and grain-derived foods, often leading to the abnormal reproduction of livestock, occasionally causing hyperestrinism in humans [6,7]. It was reported that pigs are very sensitive to ZEN, which causes swelling of the vulva and breast, vulvovaginitis, vaginal and/or rectal prolapse, interruption of pregnancy, miscarriage, and infertility in female pigs [8]. Under the condition of acute poisoning, it will cause injury to the heart, kidneys, nervous system, liver, and lungs of the animals. Hence, it is extremely important to find effective methods to remove ZEN from feedstuffs and foods.

The existing detoxification methods mainly include physical, chemical, and biological degradation methods. Compared to physical and chemical methods, the biodegradation method has the advantages of lower cost, higher efficiency, stronger specificity, no secondary pollution to feed and environment, and benefits to ecological restoration [9]. Since the 1980s, microbiologists have begun to isolate various microorganisms that can degrade ZEN around the world [8]. It was reported that over 90% of ZEN in the culture medium was degraded in 48 h by *S. cerevisiae*, which was isolated from silage [10], and 2.7 μg/mL of ZEN in the broth was completely degraded in 48 h by *S. cerevisiae*, which was isolated from grape [11]; also, ZEN was degraded 100% or 87%, especially by *B. subtilis* and *B. natto*, in 48 h [12]. However, *S. cerevisiae* and *Bacillus* were generally taken as safe potential strains in the detoxification of ZEN in Feed. ZEN was degraded 98% after incubation with *B. licheniformis* CK1 in 36 h [13]

ZEN is usually bonded specifically and hydrolyzed into less toxic products by bacteria bioactive substances. Wang et al. reported that *Bacillus subtilis* CotA laccase (BsCotA) degraded aflatoxin B1 and ZEN [14]. Yu et al. studied the active substance secreted from *Acinetobacter* sp.SM04 and found that the substance existed in the extracellular extract of liquid culture. They isolated and purified two enzymes from the liquid culture and analyzed that the toxicity of the product obtained by the degradation of ZEN was also reduced, indicating that the degradation of ZEN by these two enzymes was effective. These two enzymes were identified as peroxidase enzymes [15].

Microbial detoxification has problems such as long strain cultivation time, low degradation activity, and difficulty in removing the strain itself in a later cultivation period, and some strains are even pathogenic bacteria. Therefore, more and more studies pay attention to probiotics with the ability to degredating mycotoxins [16], and we isolated and cultivated the *Bacillus subtilis* ZJ-2019-1, which could degrade ZEN in vitro [17].

This experiment was designed to observe the poisoning symptoms of gilts and to detect the blood reproduction index and organ cell injuries in gilts that consumed diets contaminated with ZEN and investigate the detoxification effect of *Bacillus subtilis* ZJ-2019-1 by adding the fermentation broth of *Bacillus subtilis* or spraying drying fungus onto the ZEN-contaminated diet in gilts.

## 2. Results

### 2.1. Growth Performance

Throughout the 28-day feeding period, all pigs appeared healthy with no mortality. No differences were observed in average daily gain (ADG), average daily feed intake (ADFI), or feed conversion ratio (FCR, grams of feed/grams of gain) (*p* > 0.05) (Table 1) between groups, implying that after 28 days, there were no negative effects on the growth performance of gilts fed the diet contaminated with 970 μg/kg of ZEN.

### 2.2. Vulva Size and Organ Weight

**Figure 1:**
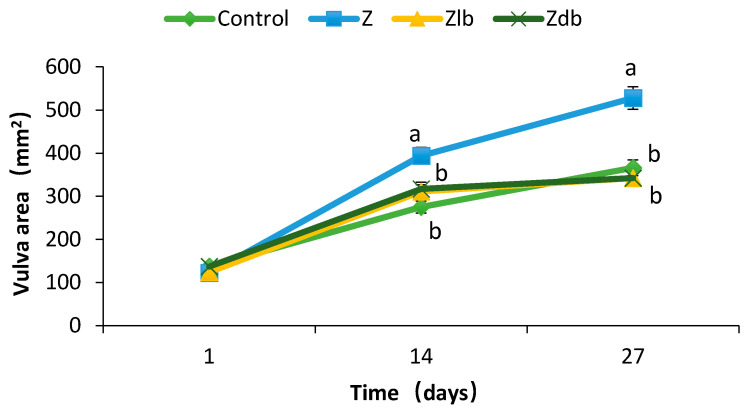
Vulva area changes in 14 days after gilts were exposed to different treatment diets. C group, control group; Z group, ZEN group; Zlb group, ZEN group diet with liquid bacteria; Zdb group, ZEN group diet with dry bacteria.

The moldy corn contaminated with ZEN negatively affected the reproductive organs of the gilts and increased the size of the vulva, while the addition of the *B. subtilis* ZJ-2019-1 strain into the diets prevented the gilts from damage caused by ZEN. There was no significant difference observed in the vulva sizes in any of the groups (*p* > 0.05), and there was no significant redness or swelling (d1–d3). Compared with the control group, the vulva size of the gilts in the Z, Zlb, and Zdb groups increased significantly (d3–d14), but the vulva size of the Zlb and Zdb gilts fed the diet containing *B. subtilis* ZJ-2019-1 exhibited a significant reduction compared with the vulva size in the Z group, returning to the normal state exhibited by the control group (d14–d27) (*p* < 0.05; Figure 1). There was no significant difference observed among the vulva sizes of the control, Zlb, and Zdb groups (*p* > 0.05), while vulva size in the Z group exhibited a significant increase compared with that of the control, Zlb, and Zdb groups (d14–d28) (*p* < 0.05).

The gilts fed the diet with 970 μg/kg of ZEN (Z group) had significantly heavier reproductive organs than those on the basic diet (control group), but the gilts fed diets containing 970 μg/kg of ZEN supplemented with *B. subtilis* ZJ-2019-1 (Zlb and Zdb group) exhibited significantly lower weights of reproductive organs (*p* < 0.05), which had no significant difference compared with the control group (Table 2). The relative weight of the liver was higher in the Z group but lower in Zlb, Zdb, and control groups (0.05 < *p* < 0.1). There were no significant changes in the relative weights of other organs (heart, kidney, and spleen) among the four groups (*p* > 0.1).

### 2.3. Serum Reproductive Hormone Level

No difference was observed in serum TP, IgG, or PROG concentration between groups (*p* > 0.05; Table 3). The concentration of serum GLDH was lower, but FSH was higher in the Z group, Zlb group, and Zdb group than in the Z group (0.05 < *p* < 0.1). Additionally, serum ALB and LH concentration had no significant difference in the C group, Zlb group, and Zdb group, but was significantly lower than in the Z group (*p* < 0.05); E2 was significantly lower in the Zlb group and Zdb group than in the C group, but higher than in the Z group (*p* < 0.05); PRL was higher in the Zlb group and Zdb group than in the C group, but lower than in the Z group (*p* < 0.05).

### 2.4. Zearalenone and Its Metabolites’ Residues

As reported in Table 4, the levels of ZEN and its metabolites expressed as the total of the free and the conjugated forms increased in the serum (*p* < 0.05) and urine (*p* < 0.05) when gilts were fed the ZEN-contaminated diet. The addition of *Bacillus subtilis* ZJ-2019-1 to the ZEN-contaminated diet was effective in reducing the concentrations of ZEN and its metabolites in the serum (0.05 < *p* < 0.1) and urine (*p* < 0.05) (Zlb and Zdb groups).

### 2.5. Pathohistological Analysis

#### 2.5.1. Histological Changes of Liver

**Figure 2:**
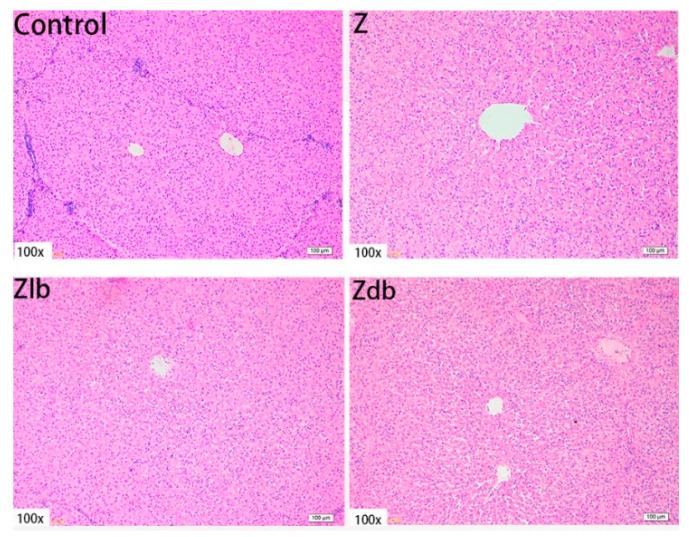
Liver of a gilt fed a basic diet (C group), ZEN-contaminated diet (Z group), ZEN-contaminated diet with liquid of *Bacillus subtilis* ZJ-2019-1 (Zlb group), or fermented–dried culture of *Bacillus subtilis* ZJ-2019-1 (Zdb group) (HE stained, 100×).

**Figure 3:**
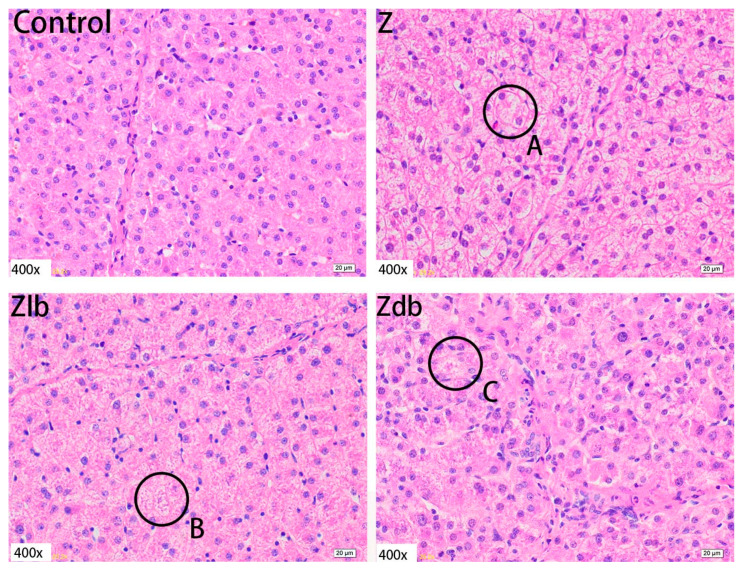
Liver of a gilt fed a basic diet (C group), ZEN-contaminated diet (Z group), ZEN-contaminated diet with liquid of *Bacillus subtilis* ZJ-2019-1 (Zlb group), or fermented–dried culture of Bacillus subtilis ZJ-2019-1 (Zdb group) (HE stained, 400×). Normal appearance of the liver was shown in control group, and ZEN caused severe cell edema and balloon-like degeneration (**A**) and granular degeneration (**B**,**C**).

In our observation, no macroscopic pathological changes in the liver were found among the treatment groups. Through pathological tissue section analysis of liver tissue, the Z group had shallower cytoplasm, shrinkage of liver lobules, increased connective tissue, and increased inflammatory cells (Figure 2) compared to the control group. However, with the addition of the bacteria, the cell damage was reduced in the Zlb and Zdb groups (Figure 2). Compared to the control group, the Z treatment group had severe cell edema and balloon-like degeneration (Figure 3A). Compared to the Z group, liver cell edema was reduced, but granular degeneration remained in the Zlb and Zdb groups (Figure 3B,C).

#### 2.5.2. Histological Changes of the Uterus

**Figure 4:**
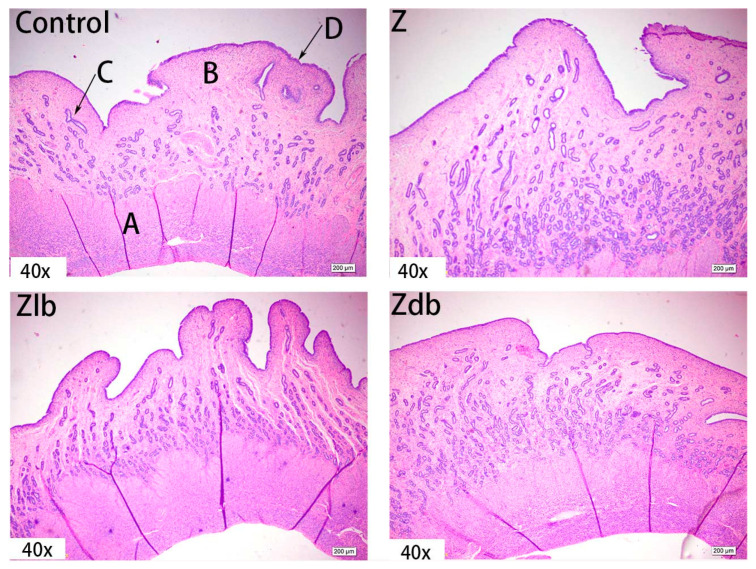
Uterus of a gilt fed a basic diet (C group), ZEN-contaminated diet (Z group), ZEN-contaminated diet with liquid of *Bacillus subtilis* ZJ-2019-1 (Zlb group), or fermented–dried culture of *Bacillus subtilis* ZJ-2019-1 (Zdb group) (HE stained, 40×). (**A**) muscle layer; (**B**) intima; (**C**) gland; (**D**) epithelium.

**Figure 5:**
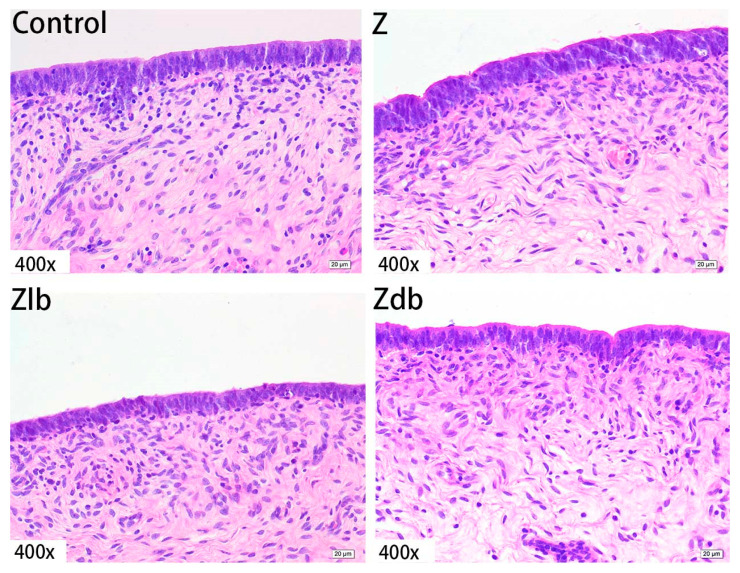
Uterine epithelial cell of a gilt fed a basic diet (C group), ZEN-contaminated diet (Z group), ZEN-contaminated diet with liquid of *Bacillus subtilis* ZJ-2019-1 (Zlb group), or fermented–dried culture of *Bacillus subtilis* ZJ-2019-1 (Zdb group) (HE stained, 400×).

**Figure 6:**
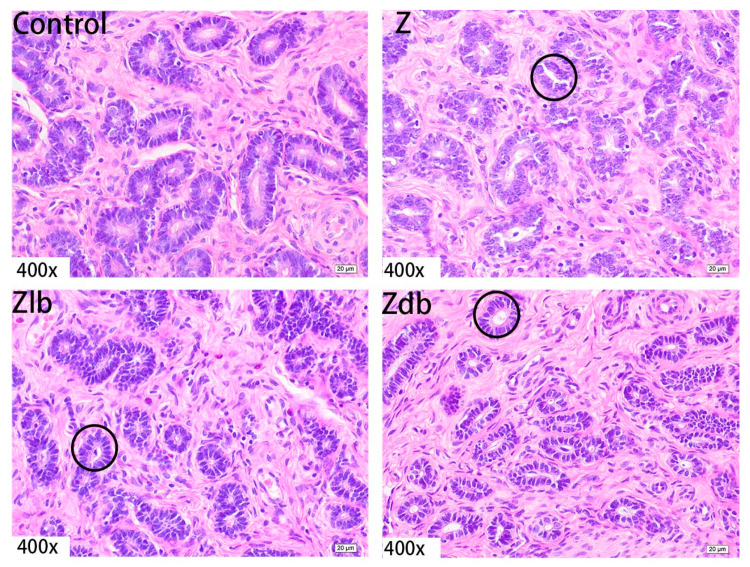
Uterine glands of a gilt fed a basic diet (C group), ZEN-contaminated diet(Z group), ZEN-contaminated diet with liquid of *Bacillus subtilis* ZJ-2019-1 (Zlb group), or fermented–dried culture of *Bacillus subtilis* ZJ-2019-1 (Zdb group) (HE stained, 400×).

Observation of the uterus revealed hypertrophy of the uterus during slaughter in the Z group. Compared to the control group, the endometrium and myometrium of the Z group were thicker, and the glands increased (Figure 4). With the bacteria degrading the ZEN in the Zlb and Zdb groups, endometrium and myometrium status were similar to the control group (Figure 4). Compared with the control group, the epithelial cells of the uterus were thickened and disorderly in the Z group, while the epithelial cells were relatively normal in the Zlb and Zdb groups (Figure 5). Compared with the control group, there were more vacuoles per unit area in the Z group, while the Zlb and Zdb groups had fewer vacuoles per unit area after adding degrading bacteria than those in the Z group (Figure 6, shown in black circles). The thickness of the endometrium and myometrium of the uterus was higher in the Z group than in the control group (*p* < 0.05), but there was no difference between the Zlb and Zdb groups and the control group (Table 5).

#### 2.5.3. Histological Changes of Ovaries

**Figure 7:**
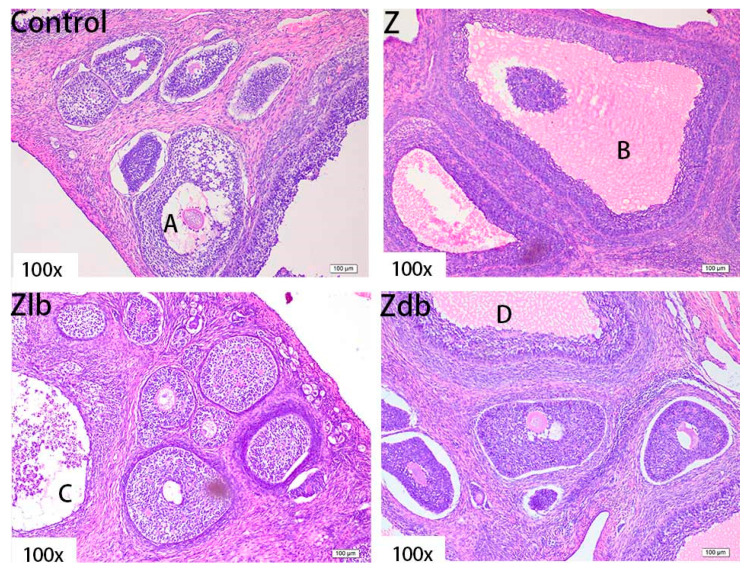
Ovaries of a gilt fed a basic diet (C group), ZEN-contaminated diet (Z group), ZEN-contaminated diet with liquid of *Bacillus subtilis* ZJ-2019-1 (Zlb group), or fermented–dried culture of *Bacillus subtilis* ZJ-2019-1 (Zdb group) (HE stained, 100×) (**A**): mature follicle; (**B**–**D**): abnormally growing follicles.

There was oocyte vacuolization in the Z group (Figure 7B), the granulosa cell layer did not detach from the interstitium, and they became abnormally growing follicles. There were some abnormal growth follicles (Figure 7C,D) in the Zlb and Zdb groups, but the size of the growth follicles became smaller and the granular layer retreated, which was similar to the control group. Although the degree of poisoning in the ovary was reduced in the Zlb and Zdb groups, the ovum disappeared, of which the damage was irreversible.

## 3. Discussion

In the present study, a diet contaminated with 970 μg/kg of ZEN had no negative effects on the growth performance of gilts fed the basal diet, which was consistent with the findings of other researchers, where growth performance among treatments was also not affected by ZEN. Our results, combined with the report that gilts fed varying amounts of dietary ZEN (1.0, 2.0, 3.0 mg/kg of diet) grew similarly with no differences in feed intake, suggests that short-term consumption of a low dose of ZEN does not negatively affect the growth performance of gilts [18]. Zhao et al. found that the production performance of pigs was not significantly affected after adding *Bacillus subtilis* ANSB01G to naturally moldy diets [19]. It is possible that the concentration of ZEN added to the diet was not enough to retard pig growth.

Vulva swelling and rectal and vaginal prolapse are the most significant symptoms of ZEN poisoning in piglets [20]. The organ index can reflect the health of the organ under certain circumstances. The toxicity of ZEN can also be expressed by increasing the organ index of the sow [20]. Zhao et al. showed an increase in the area of the vulva and a significantly increase in the reproductive organs index but has no significant effect on other organs (heart, liver, spleen, kidney) after feeding ZEN-contaminated diets to young sows (the ZEN content was 238.57 μg/kg) [19]. However, the redness and swelling phenomenon was improved after the addition of *Bacillus subtilis* ANSB01G [19]. Additionally, the reproductive organs index decreased. The combination of probiotics with cell-free extracts of *Aspergillus oryzae* alleviated the red and swelling vulva symptoms [21]. This experiment found that a ZEN content of 970 μg/kg in the diet of gilts significantly increased the area of the vulva and reproductive organs index (uterine ovary). It also caused redness and swelling of the vulva. After adding the degrading bacteria ZJ-2019-1 to the ZEN mildew diet, it was found that on day 27, the vaginal area reached a predetermined level, which alleviated the symptoms of red and swollen vaginas in gilts. Additionally, the reproductive organs index decreased. This suggests that *B. subtilis* ZJ-2019-1 degrades part of ZEN in the feed, thereby reducing the effect of ZEN on gilts.

It is found that ZEN is a non-sterol estrogen, which has a special affinity with the estrogen receptor in the body, which leads to hyperestrogenism [22,23]. It was observed that ZEN significantly reduced the content of luteinizing hormone, estradiol, follicle-stimulating hormone, and progesterone in Tibetan pig serum but has no significant effect on testosterone content [24]. Zhao reported that after young sows ingested ZEN-contaminated diets, serum prolactin levels increased, but there was no significant effect on estradiol, luteinizing hormone, and follicle-stimulating hormone levels. When they added the degrading bacteria, the prolactin content in the serum was consistent with the control group [19]. The present results indicated that prepubertal gilts exposed to ZEN without *B. subtilis* ZJ-2019-1 exhibited an increased level of PRL in serum, promoting a significant reduction in the ratio of LH and E2. Adding *B. subtilis* ZJ-2019-1 to diets contaminated with ZEN can maintain LH content in the serum of gilts, which is close to that of the control group. Compared with the Z group who consumed contaminated diets, serum estradiol levels increased, and prolactin levels decreased. This indicates that *B. subtilis* ZJ-2019-1 has a protective effect on ZEN toxicosis symptoms in gilts.

The mycotoxins and their metabolites in animal blood, bile, milk, urine, and feces can be used as biomarkers of mycotoxins. The frequency and level of animal exposure to mycotoxins can be evaluated by measuring their contents in animals [25,26]. ZEN produces different metabolites in different animals and is mainly converted into α-ZOL and a small amount of β-ZOL in pigs [27]. In the present study, adding 970 μg/kg of ZEN to the diet significantly increased the serum and urine contents of ZEN, α-ZOL, and β-ZOL in gilts (*p* < 0.05). After *Bacillus subtilis* ZJ-2019-1 was added to the contaminated diet ingested by gilts, the content of ZEN and its main metabolites decreased in serum and urine, and the content decreased significantly in urine (*p* < 0.05) and remained at the control group level, which suggested that *Bacillus subtilis* ZJ-2019-1 degraded part of the ZEN in the feed, thereby reducing the residual amount of ZEN and its main metabolites in serum and urine.

ZEN is mainly metabolized in the liver and excreted in urine or feces in the form of glucuronide after multiple hepatic and enterocirculations, so it might cause certain damage to the liver during the ZEN degradation. At the same time, ZEN also causes great damage to reproductive cells, binding to estrogen receptors and exhibiting estrogenic activity, causing reproductive hormone disorders in animals and destroying the reproductive system of animals. ZEN can cause swelling of the uterus, dysplasia of follicles in the ovaries, and even miscarriage [28]. Microbial additives have been reported for use in mycotoxin decontamination feeding strategies. Studies have shown that the addition of yeast products to basal diets can reduce the effects of mycotoxins on pig growth and health [29]. It was reported that the morphological observation of the uterus and ovaries of gilts by ZEN and found that the treatment group with a high amount of ZEN (2 mg/kg) had the most significant effect on the morphology of the uterus. The main change was swelling of the uterus. The endometrium and myometrium become thicker, and the glands and blood vessels of the endometrium increase and become longer. These changes in tissue morphology are consistent with the increase in the volume and weight of the uterus [30]. Liver cells presented symptoms of swelling and inflammation, and lymphocyte infiltration can be effectively ameliorated by adding *B. subtilis* ANSB01G to diets naturally contaminated with ZEN [19]. In our experiment, adding 970 μg/kg ZEN to the diet can cause liver tissue to become shallower, liver lobules shrink, connective tissue increases, inflammatory cells increase, and hepatocyte edema. The endometrium and myometrium are significantly thickened. The glands increase, and vacuolization occurs. Meanwhile, abnormal growth follicles appear in the ovarian tissue; the follicles are very large, but the granular layer is not separated from the interstitium. In combination with the above reproductive hormones, the reproductive hormone content in the serum of the gilts fed ZEN-contaminated diets is reduced, but the follicles are still developing, indicating that ZEN has an estrogen-like effect and promotes the growth of the follicles, but the ovum disappears, and the follicles cannot be ruptured, so the female animal is only in estrus and not fertility. After adding degrading bacteria to the contaminated diet, the damage of each tissue cell was slightly improved, indicating that *B. subtilis* ZJ-2019-1 degraded part of ZEN in the feed, but for the disappearance of the ovum in the ovary, this kind of cell damage is irreversible.

Though mycotoxin adsorbents are used worldwide to control mycotoxins in feed, these adsorbents merely shift the mycotoxins to the surrounding region, contributing to the contamination of the environment [19]. Biodegradation is known to be a highly efficient, specific, and environmentally friendly method for the treatment of mycotoxins in both foods and feeds [31]. We found that a strain of *Bacillus subtilis* ZJ-2019-1 is very effective in detoxifying ZEN, which degraded 10 mg L^−1^ of zearalenone within 48 h in vitro [17]. In addition, it has been confirmed by our group that the mechanism by which *B. subtilis* ZJ-2019-1 degrades zearalenone is mainly biodegradation, not cell adsorption [17]. Hence, *B. subtilis* ZJ-2019-1 can be used as a potential tool for the detoxification of ZEN in animal feeds and feedstuffs.

## 4. Conclusions

The present study demonstrates that a diet naturally contaminated with ZEN at 970 μg/kg diet could cause severe reproductive toxicity to gilts, but *B. subtilis* ZJ-2019-1 could ameliorate the toxic effects effectively. *B. subtilis* ZJ-2019-1 has great potential industrial applications. Further studies on cellular and molecular mechanisms will be needed to thoroughly understand the mechanism *B. subtilis* ZJ-2019-1 in alleviating the reproductive toxicity of ZEN in vivo.

## 5. Materials and Methods

### 5.1. Bacterial Preparation

A single *Bacillus subtilis* ZJ-2019-1 colony was picked and inoculated in LB medium containing 180 mL of sterile liquid at 37 °C, at 200 rpm, for 24 h. The numbers of viable bacteria were 7.2 × 10^10^ CFU/mL in the fermentation broth, and half of it was used to mix with the ZEN-contaminated feed, and half was made into dry bacterial preparation by spray drying with soluble starch as protector, in which the numbers of viable bacteria were 1 × 10^10^ CFU/g.

### 5.2. Preparation of Zearalenone-Contaminated Diet

We chose natural moldy corn contaminated by toxin detection analysis and selected moldy corn contaminated only by ZEN, with ZEN content of 1158.67 μg/kg as the experimental materials; the ZEN content was lower than the detection limits, and the LOD of the dietary ZEN was 10–500 μg/kg.

Four diets were prepared as follows: a basic diet formulated according to NRC was used as diet in the C group; Zen diet was the same formula as the basic diet, but the corn was replaced by the one contaminated with ZEN (final concentrations of 970 μg/kg of ZEN); Zlb diet was fed on ZEN diet with liquid bacteria (30 mL/head/d of bacterial liquid of *B. subtilis* ZJ-2019-1, 7.2 × 10^10^ CFU/mL), and Zdb group was fed on Z group diet with dry bacteria (2 kg/T of fermented–dried culture of *B. subtilis* ZJ-2019-1). The mycotoxin contamination, ZEN, deoxynivalenol (DON), aflatoxin (AFT), and ochratoxin (OAT), in the feed was determined according to the method (HPLC) of Chinese Certifications GB/T23504, GB/T 18979-2003, GB/T23503-2009, and GB/T23502-2009, respectively.

Dietary ZEN determination. Feed samples were milled, and 25 g of fine powder was taken and mixed with 100 mL of 70:30 acetonitrile/distilled water (*v*/*v*) solution for 3 min and then filtered. The filtrate was diluted with PBS solution (pH 7.4) and then filtered by a microfiber filter paper. A total of 10 mL diluted filtrate was passed through a ZEN immunoaffinity column (Aokinlmmuno Clean CF ZEA, Aokin, Germany) at a flow rate of 1 mL/min. The column was washed with distilled water at a flow rate of 3 mL/min, and then ZEN was eluted with 2 mL of methanol. Then, 20 µL of eluate was determined by HPLC system equipped with a ZORBAX SB-C18 column (150 mm × 4.6 mm × 5 µm, Agilent Technology Inc., Palo Alto, CA, USA) and a fluorescence detector (RF-20A, Shimadzu, Japan) set at excitation and emission wavelengths of 274 and 440 nm, respectively, and a solution of acetonitrile:distilled water:methanol (46:46:8, *v*/*v*/*v*) serving as mobile phase.

### 5.3. Animal Management

A total of 24 female gilts (Landrace × Yorkshire × Duroc, 20.67 ± 3.65 kg BW, 60.30 ± 5.63 d old) were randomly assigned to four groups, six replicates per group, and one gilt per replicate. The control group was fed a basic diet with normal corn (C group), Z group was fed the diet in same formula as the basic diet, but with the normal corn replaced by the one contaminated with ZEN (final concentrations of 970 μg/kg of ZEN), Zlb group was fed the Z group diet with liquid bacteria (30 mL/head/d of bacterial liquid of *B. subtilis* ZJ-2019-1, 7.2 × 10^10^ CFU/mL), and Zdb group was fed the Z group diet with dry bacteria (2 kg/T of fermented–dried culture of *B. subtilis* ZJ-2019-1). The gilts were fed at 8:00 and 17:00 and had free access to fresh and clean water. The feeding adaption period was 7 days, and the experiment period was 21 days. Representative feed samples were taken at the beginning and end of feeding for nutrient analyses; the daily intake, initial BW, and final BW were recorded. Nutrient contents in feed were, respectively, determined according to Chinese standard methods for the determination of crude protein, crude fat, crude fiber, moisture and other volatile matter content, calcium, and phosphorus in feeds (GB/T 6432-1994, GB/T 6433-2006, GB/T 6434-2006, GB/T 6435-2006, GB/T 6436-2002, and GB/T 6437-2002; Table 6).

The experimental protocol was approved by the Laboratory Animal Ethics Committee Feed Research Institute, Chinese Academy of Agricultural Sciences (the approval number is AEC-CAAS-20180915, approval date: 15 September 2018), which was performed in accordance with animal welfare practices and procedures followed the Guidelines for Animal Experiments by the Ministry of Science and Technology (2006, Beijing, China).

### 5.4. Vulva Measurements, Sample Collection, and Analysis

Vulva length and width were measured at days 1, 14, and 27, and the vulva area was calculated as an approximately oval shape ((π × vulva length × vulva width)/4) according to method reported by Zhao et al. Pigs (20.67 ± 3.65 kg BW) were fasted for 12 h at the end of the experimental period. Blood samples were obtained by anterior vena cava puncture using 10 mL anticoagulant free vacuum tubes and then centrifuged at 3000× *g* for 15 min, and serum was collected for subsequent analysis. Then, pigs were euthanized. All animal handling and treatment procedures were in accordance with the Laboratory Animal Ethics Committee of FRI, CAAS mentioned above. Heart, lung, liver, kidney, spleen, and reproductive organs (ovary + uteri) were examined for their general appearance and then isolated and weighed, and their weights were calculated based on relative body weight (g/kg). Ovary, uterus, and liver samples were collected and fixed in a 10% neutral buffered formalin solution immediately following the end of the experiment.

Serum total protein (TP): total protein kit (biuret colorimetric method, Shanghai Kehua Bio-engineering Co., Ltd., Shanghai, China), albumin (ALB): albumin kit (bromocresol green method, Shanghai Kehua Bio-engineering Co., Ltd.), immunoglobulin (IgG): porcine immunoglobulin IgG/A/M determination kit (Shanghai Kehua Bio-engineering Co., Ltd.) and glutamate dehydrogenase (GLDH): glutamate dehydrogenase kit (spectrophotometry, Nanjing Jiancheng Bioengineering Institute, Nanjing, China); and reproductive hormone indicators, follicle-stimulating hormone (FSH), luteinizing hormone (LH), estradiol (E2), prolactin (PRL), and progesterone (P) were, respectively, measured by ELISA quantification kits (Nanjing Jiancheng Bioengineering Institute, Nanjing, China).

Serum/urine ZEN and its main metabolites were analyzed according to the method reported by Kongkapan et al. with modification [32], briefly described as follows. Firstly, a quantity of 0.5 mL of a serum/urine sample was mixed with 5 μL of 1 mg/L ZEN internal standard (40 μL of 25 μg/L ^13^C-ZEN dissolved in 960 μL of acetonitrile), 1.5 mL of 0.2 M ammonium acetate, and 20 μL of glucuronide/sulfatase, and then incubated in gas bath shaker at 55 °C for 2 h. Secondly, 6 mL of 0.1% formic acid acetonitrile was added to the zymolytic solution, then the solution was placed on vortex at 2000 rpm for 1 min, then kept at 4 °C for 20 min, and then 0.4× *g* sodium chloride and 1× *g* anhydrous sulfuric acid magnesium were added, then the solution was vortexed at 2000 rpm for 1 min, and centrifuged at 8000 r/min for 5 min. After being deposited for a while, 6 mL of the upper layer solution (only the upper layer solution to avoid taking the lower water phase) was moved into a 10 mL stoppered plastic centrifuge tube, 500 mg of QuECHERs purification material (200 mg of C18, 100 mg of PSA, and 100 mg of A-AL) was added, along with 200 mg of anhydrous magnesium sulfate, and the solution was placed on vortex at 2000 rpm for 1 min and centrifuged at 8000 r/min for 5 min. Additionally, 4.8 mL of the filtrate was accurately measured and placed in a 10 mL plastic centrifuge tube. It was evaporated in a vacuum concentrator at 60 °C and 1500 r/min and then dissolved with 0.2 mL of 0.1% formic acid/methanol/water (0.1/50/49.9, *v*/*v*/*v*) [24], and the concentrations of ZEN and its metabolites, α-zearalenol and β-zearalenol, were determined by high-performance liquid chromatography (XEVO TQ-S, Waters, Milford, MA, USA) equipped with a ZORBAX SB-C18 column (150 mm × 4.6 mm × 5 µm, Agilent Technology Inc., Palo Alto, CA, USA) and a fluorescence detector (RF-20A, Shimadzu, Japan) set at excitation and emission wavelengths of 274 and 440 nm, respectively, and a solution of acetonitrile/distilled water/methanol (46/46/8, *v*/*v*/*v*) serving as mobile phase.

The ovary, uterus, and liver in the tissue fixative were taken out, embedded in paraffin, and cut to acquire sections of 5 μm thickness using routine histological techniques. The sections were fixed on glass slides and stained with hematoxylin and eosin (H&E). The slides were examined with an optical microscope (Nikon ELIPSE 80i, Kawasaki, Japan) to evaluate the morphologic changes.

### 5.5. Statistical Analysis

The experimental design was completely randomized using four treatments and six replicates, with each animal considered a random unit. The statistical analysis was conducted using Statistical Product and Service Solutions software (SPSS Inc., Chicago, IL, USA), and the data are expressed as the mean the standard error of the mean. Because the data were normally distributed, all the indices were analyzed using ANOVA and Duncan’s multiple range tests. Differences are considered significant when *p* < 0.05, and trends are noted when 0.05 < *p* < 0.1. Usually individual comparison of means (Duncan’s test) is performed when ANVOA reveals significant difference among groups, and groups that are significantly different are marked with different letter.

## Figures and Tables

**Table 1:** Effects of *Bacillus subtilis* ZJ-2019-1 on the performance of gilts exposed to ZEN.

Items	C Group	Z Group	Zlb Group	Zdb Group	*p* Value
Initial body weight, kg	20.60 ± 2.58	21.00 ± 5.09	21.72 ± 2.79	19.36 ± 4.38	0.805
Final body weight, kg	30.60 ± 2.54	31.32 ± 4.90	32.96 ± 2.67	27.88 ± 7.85	0.461
ADG, kg/d	0.34 ± 0.00	0.36 ± 0.02	0.39 ± 0.04	0.29 ± 0.16	0.366
ADFI, kg/d	0.75 ± 0.05	0.77 ± 0.053	0.79 ± 0.10	0.62 ± 0.28	0.342
FCR, kg/kg	2.17 ± 0.14	2.16 ± 0.09	2.03 ± 0.05	2.24 ± 0.18	0.302

ADG, average daily gain; ADFI, average daily feed intake; FCR, feed conversion ratio; C group, control group; Z group, ZEN group; Zlb group, ZEN group diet with liquid bacteria; Zdb group, ZEN group diet with dry bacteria. Values are expressed as means ± SD, differ significantly at *p* < 0.05, and trends are noted when 0.05 < *p* < 0.1.

**Table 2:** Effects of *Bacillus subtilis* ZJ-2019-1 on the relative weights of organs of gilts exposed to ZEN.

Items	C Group	Z Group	Zlb Group	Zdb Group	*p* Value
Heart	4.40 ± 0.34	4.25 ± 0.34	4.22 ± 0.34	3.69 ± 0.72	0.32
Liver	24.89 ± 0.19 ab	28.17 ± 3.58 a	25.68 ± 2.17 ab	20.24 ± 3.97 b	0.07
Spleen	1.69 ± 0.33	1.59 ± 0.11	1.70 ± 0.17	1.55 ± 0.61	0.95
Kidney	4.02 ± 0.41	3.62 ± 0.46	4.17 ± 0.76	3.52 ± 1.45	0.77
Lung	14.01 ± 2.56	16.70 ± 1.27	17.98 ± 3.18	13.64 ± 1.71	0.13
Reproductive organs *	1.51 ± 0.27 b	2.52 ± 0.34 a	1.09 ± 0.45 b	1.23 ± 0.67 b	0.02

C group, control group; Z group, ZEN group; Zlb group, ZEN group diet with liquid bacteria; Zdb group, ZEN group diet with dry bacteria. * The weight of ovary and uterus was weighed and recorded as reproductive organs. Values are expressed as means ± SD, differ significantly at *p* < 0.05, and trends are noted when 0.05 < *p* < 0.1. Usually individual comparison of means (Duncan’s test) is performed when ANVOA reveals significant difference among groups, and groups that are significantly different are marked with different letter.

**Table 3:** Effects of *Bacillus subtilis* ZJ-2019-1 on serum biochemical index and reproductive hormones of gilts exposed to ZEN.

Items	C Group	Z Group	Zlb Group	Zdb Group	*p* Value
TP, g/L	60.66 ± 2.02	60.85 ± 2.38	62.58 ± 1.44	63.97 ± 2.23	0.11
ALB, g/L	34.07 ± 0.93 a	30.76 ± 1.86 b	32.46 ± 1.03 ab	33.06 ± 0.60 a	0.03
IgG, g/L	8.30 ± 0.11	8.21 ± 0.03	8.28 ± 0.08	8.32 ± 0.04	0.30
GLDH, U/mL	18.33 ± 0.22	19.29 ± 0.45	18.66 ± 0.54	18.37 ± 0.21	0.06
FSH, mIU/mL	16.23 ± 1.02	14.97 ± 0.38	16.09 ± 1.16	16.59 ± 0.49	0.08
LH, mIU/mL	12.99 ± 1.01 a	11.46 ± 0.84 b	12.10 ± 2.07 a	12.08 ± 0.22 a	0.04
E2, pg/mL	53.80 ± 6.04 a	35.85 ± 8.05 c	36.48 ± 3.56 b	37.22 ± 7.73 b	0.001
PRL, mIU/L	68.46 ± 9.83 c	109.88 ± 9.79 a	78.32 ± 7.56 b	78.24 ± 7.97 b	0.00
PROG, ng/mL	4.10 ± 3.68	3.24 ± 0.68	3.10 ± 0.35	3.55 ± 0.86	0.85

TP, total protein; ALB, albumin; GLDH, glutamine dehydrogenase; FSH, follicle-stimulating hormone; LH, luteinizing hormone; E2, estradiol; PRL, prolactin; PROG, progesterone. C group, control group; Z group, ZEN group; Zlb group, ZEN group diet with liquid bacteria; Zdb group, ZEN group diet with dry bacteria. Values are expressed as means ± SD, differ significantly at *p* < 0.05, and trends are noted when 0.05 < *p* < 0.1. Usually individual comparison of means (Duncan’s test) is performed when ANVOA reveals significant difference among groups, and groups that are significantly different are marked with different letter.

**Table 4:** Effects of *Bacillus subtilis* ZJ-2019-1 on the concentration of ZEN and its metabolites in serum/urine of gilts exposed to ZEN.

**Items, ng/mL**	**Serum**				
	**C Group**	**Z Group**	**Zlb Group**	**Zdb Group**	***p* Value**
ZEN	0.12 ± 0.05 b	2.94 ± 1.18 a	2.80 ± 1.28 a	2.88 ± 0.55 a	0.002
α-ZOL	0 b	2.42 ± 0.54 a	2.24 ± 0.63 a	1.69 ± 1.57 a	0.009
β-ZOL	0	0	0	0	1
ZEN+α-ZOL+β-ZOL	0.12 ± 0.05 b	5.36 ± 1.37 a	5.04 ± 1.46 a	4.57 ± 1.21 a	<0.0001
**Items, ng/mL**	**Urine**				
	**C Group**	**Z Group**	**Zlb Group**	**Zdb Group**	***p* Value**
ZEN	11.70 ± 0.34 b	139.01 ± 5.33 a	13.13 ± 3.43 b	21.07 ± 2.49 b	<0.0001
α-ZOL	6.50 ± 0.57 b	59.92 ± 0.61 a	6.92 ± 0.29 b	7.40 ± 0.54 b	0.04
β-ZOL	2.28 ± 0.22 b	11.89 ± 1.25 a	0 b	1.11 ± 0.157 b	0.01
ZEN+α-ZOL+β-ZOL	20.48 ± 0.14 b	210.82 ± 3.18 a	20.05 ± 3.72 b	29.58 ± 7.61 b	0.002

ZEN, zearalenone; α-ZOL, α- Zeralenol; β-ZOL, β- Zeralenol. C group, control group; Z group, ZEN group; Zlb group, ZEN group diet with liquid bacteria; Zdb group, ZEN group diet with dry bacteria. LOD was 0.05 ng/mL, and LOQ was 100 ng/mL. Values are expressed as means ± SD, differ significantly at *p* < 0.05, and trends are noted when 0.05 < *p* < 0.1. Usually individual comparison of means (Duncan’s test) is performed when ANVOA reveals significant difference among groups, and groups that are significantly different are marked with different letter.

**Table 5:** Effects of *Bacillus subtilis* ZJ-2019-1 on endometrial thickness and myometrial thickness of gilts when exposed to ZEN.

Items	C Group	Z Group	Zlb Group	Zdb Group	*p* Value
Epithelium thickness, μm	1103.42 ± 112.24 b	1574.15 ± 300.40 a	1127.37 ± 189.61 b	1218.46 ± 304.97 b	0.03
Muscle thickness, μm	873.69 ± 55.18 b	1102.22 ± 148.33 a	936.32 ± 137.74 ab	815.24 ± 229.85 b	0.05

C group, control group; Z group, ZEN group; Zlb group, ZEN group diet with liquid bacteria; Zdb group, ZEN group diet with dry bacteria. Values are expressed as means ± SD, differ significantly at *p* < 0.05, and trends are noted when 0.05 < *p* < 0.1. Usually individual comparison of means (Duncan’s test) is performed when ANVOA reveals significant difference among groups, and groups that are significantly different are marked with different letter.

**Table 6:** The ingredients and nutritional components of the basic diet.

Ingredients	%	Nutrition Levels ^b^	
Maize	51.00	DE (MJ/kg)	12.79
Wheat bran	5.00	Crude protein (%)	18.83
Whey powder	5.50	Lysine (%)	1.18
Soybean meal	14.00	Methionine (%)	0.43
Cottonseed meal	6.00	Thionine (%)	0.68
Corn germ meal	3.00	Threonine (%)	0.81
Puffed full-fat soybeans	5.00	Tryptophan (%)	0.23
Fish meal	5.50	Calcium (%)	0.72
Stone powder	0.80	Total phosphorus (%)	0.65
Soybean oil	2.50		
Salt	0.30		
Dicalcium phosphate	0.40		
Premix ^a^	1.00		

^a^ Provided per kg of diet: vitamin A, 14 000 IU; vitamin D3, 2200 IU; vitamin E, 16 IU; vitamin K3, 2.2 mg; vitamin B1, 2.3 mg; vitamin B2, 4.5 mg; vitamin B5, 13 mg; niacin, 26 mg; pantothenic acid, 14 mg; folacin, 0.6 mg; copper, 60 mg; iron, 140 mg; zinc, 60 mg; manganese, 20 mg; selenium, 0.40 mg; and iodine, 0.21 mg. ^b^ Nutrient levels were measured values.

## Abbreviations

ZEN	Zearalenone
α-ZOL	α-Zearalenol
β-ZOL	β-Zearalenol
ADG	Average Daily Gain
ADFI	Averge Daily Feed Intake
TP	Total protei
ALB	Albumin
E2	Estradiol
FCR	Feed Coversion Rate
FSH	Follicle-Stimulating Hormone
GLDH	Glutamate dehdrogenase
IgG	Immunoglobulin G
LH	Luteinizing Hormone
PRL	Prolactin
PROG	Progesterone
HPLC	High Performance Liquid Chromatography
PSA	Primary Secondary Amine
